# NK Cells of Kidney Transplant Recipients Display an Activated Phenotype that Is Influenced by Immunosuppression and Pathological Staging

**DOI:** 10.1371/journal.pone.0132484

**Published:** 2015-07-06

**Authors:** Ulrike Hoffmann, Christine Neudörfl, Kerstin Daemen, Jana Keil, Maja Stevanovic-Meyer, Frank Lehner, Hermann Haller, Cornelia Blume, Christine S. Falk

**Affiliations:** 1 Institute of Transplant Immunology, IFB-Tx, Hannover Medical School Hannover, Hannover, Germany; 2 Department of Visceral and Transplant Surgery, Hannover Medical School, Hannover, Germany; 3 Department of Nephrology and Hypertension, Hannover Medical School, Hannover, Germany; 4 Institute of Technical Chemistry, Leibniz University Hannover, Hannover, Germany; 5 DZIF, German Center for Infectious Diseases, Hannover / Braunschweig, Germany; Karolinska Institutet, SWEDEN

## Abstract

To explore phenotype and function of NK cells in kidney transplant recipients, we investigated the peripheral NK cell repertoire, capacity to respond to various stimuli and impact of immunosuppressive drugs on NK cell activity in kidney transplant recipients. CD56^dim^ NK cells of kidney transplanted patients displayed an activated phenotype characterized by significantly decreased surface expression of CD16 (p=0.0003), CD226 (p<0.0001), CD161 (p=0.0139) and simultaneously increased expression of activation markers like HLA-DR (p=0.0011) and CD25 (p=0.0015). Upon *in vitro* stimulation *via* Ca^++^-dependent signals, down-modulation of CD16 was associated with induction of interferon (IFN)-γ expression. CD16 modulation and secretion of NFAT-dependent cytokines such as IFN-γ, TNF-α, IL-10 and IL-31 were significantly suppressed by treatment of isolated NK cells with calcineurin inhibitors but not with mTOR inhibitors. In kidney transplant recipients, IFN-γ production was retained in response to HLA class I-negative target cells and to non-specific stimuli, respectively. However, secretion of other cytokines like IL-13, IL-17, IL-22 and IL-31 was significantly reduced compared to healthy donors. In contrast to suppression of cytokine expression at the transcriptional level, cytotoxin release, i.e. perforin, granzyme A/B, was not affected by immunosuppression *in vitro* and *in vivo* in patients as well as in healthy donors. Thus, immunosuppressive treatment affects NK cell function at the level of NFAT-dependent gene expression whereby calcineurin inhibitors primarily impair cytokine secretion while mTOR inhibitors have only marginal effects. Taken together, NK cells may serve as indicators for immunosuppression and may facilitate a personalized adjustment of immunosuppressive medication in kidney transplant recipients.

## Introduction

The role of natural killer (NK) cells in solid organ transplantation is discussed controversially [1]. On one hand, NK cells were shown to be involved in graft rejection as they express the FcγRIII (CD16) and bind to donor-specific antibodies (DSA) directed against allogeneic HLA molecules expressed by the grafted organ [2]. Furthermore, NK cell-derived signatures were identified in biopsies of kidney transplant recipients (KTR) with antibody-mediated rejection [3]. On the other hand, NK cell signatures have been identified in peripheral blood of operationally tolerant patients after kidney or liver transplantation indicating an involvement in tolerogenic mechanisms [4,5]. These divergent observations argue for a context-dependent involvement of NK cells in either rejection or tolerance induction.

NK cells belong to the recently defined group of innate lymphoid cells (ILC) that primarily mediate host defense against pathogens by their cytotoxic activity in combination with cytokine production [6]. Peripheral blood NK cells can be divided into two subsets differing in phenotype and function [7]. CD56^dim^ NK cells are characterized by low CD56 and high expression of CD16, the scavenger receptor CD6, killer-Immunoglobuline-like receptors (KIR) and the senescence marker KLRG1 and represent mature NK cells. CD56^bright^ NK cells lack CD16, CD6 and KIR but highly express CD56, the inhibitory heterodimer CD94/NKG2A and CD62L which assigns them to an immature status [8]. In contrast, natural cytotoxicity receptors (NCR), NKG2D, CD161 and others are equally expressed on both subsets. These different phenotypic features are associated with different effector functions. CD56^dim^ NK cells mediate cytotoxicity after various triggers which is controlled by inhibitory receptors: encounter of KIR with self HLA class I molecules leads to receptor-mediated inhibition *via* blocking of activating signaling pathways by recruitment of phosphatases [9]. In the absence of self HLA class I molecules, engagement of activating receptors delivers positive signals *via* CD16, NKp46, NKp30, NKG2D and CD226 (DNAM-1) that induce degranulation and secretion of pro-inflammatory cytokines. The magnitude of cytokine secretion depends on the stimulus whereby target cell recognition is superior to cytokine stimulation based upon the involvement of intracellular signaling *via* several activating receptors instead of Jak/STAT signaling *via* cytokine receptors [10]. CD56^bright^ NK cells have been described to produce higher amounts of cytokines like IFN-γ in response to stimulation with cytokines such as IL-2, IL-12 or IL-15 and to exert little cytotoxicity compared to CD56^dim^ NK cells [11]. The role of these NK cell subsets in kidney transplantation (KTx) has not been clarified yet, although recent studies revealed that a KIR-ligand mismatch between donor HLA class I molecules and recipient KIR repertoire has no impact on allograft outcome [12]. In a recent study, however, the presence of the KIR2DS3 gene in KTx recipients was associated with better graft function in the absence of the HLA class I ligand in the donor [13]. In addition, lower NK cell numbers with increased proportions of CD56^bright^ NK cells were identified in DSA-positive kidney transplanted patients [14].

Information regarding the influence of immunosuppressive drugs on NK cell activity in kidney recipients is rather limited compared to T cells that are supposed to represent the major targets for immunosuppressive drugs. Most of the knowledge regarding nuclear factor of activated T cells (NFAT) and mammalian target or rapamycin (mTOR) signaling pathways has emerged from studies addressing T cells expressing several NFAT family members with NFAT1, 2 and 4 as most abundantly expressed members [15] and NFAT2 responsible for sustained signal transduction by its inducible isoform [16]. Compared to T cells, little is known about the regulation of NFAT induction in NK cells, especially not with respect to their susceptibility to calcineurin inhibitor (CNI) treatment after KTx and some studies even suggest that NK cells might be refractory to CNI treatment [1,17].

In our previous study, we could show that the NK cell phenotype, especially of the CD56^dim^ subset, is significantly altered in KTx recipients [18]. Compared to healthy donors, NK cells of patients displayed significantly less CD16 and CD6 surface expression which correlated to the immunosuppressive treatment whereby patients with Tacrolimus-based drug regimen, usually containing co-medication with MPA and corticosteroids, displayed the strongest phenotypic changes. This observation is likely due to the triple immunosuppression comprising tacrolimus, mycophenolate and corticosteroids which represents the most effective immunosuppressive regimen with tacrolimus enhancing mycophenolate drug levels *via* increased enterohepatic recirculation [19]. Most importantly, the presence of donor-specific antibodies (DSA) was associated with lower NK cell numbers in peripheral blood. Since we found these alterations in the blood of kidney transplanted patients, it was the aim of this study to investigate the functional consequences of these phenotypical alterations and to unravel differences between immunosuppressive drugs with respect to long-lasting functional impairment of NK cells, especially in terms of cytokine production.

Here, we demonstrate that in addition to CD16 and CD6, other receptors like CD226 and CD161 were down-regulated on NK cells of KTx patients which was associated with increased expression of activation markers such as CD25, CD69 and HLA-DR. While highest HLA-DR expression was detected on CD56^dim^ NK cells at 3 months after transplantation and declined at 9 months to normal levels, CD25 and CD69 expression did not show this time dependent up-regulation. Pathological staging of kidney biopsies was associated with an altered NK cell phenotype, i.e. CD226 expression was significantly reduced on CD56^dim^ NK cells of patients with T cell-mediated rejection (TCMR). HLA-DR expression was significantly lower on CD56^dim^ NK cells of patients with antibody-mediated rejection (AMR) compared to patients with borderline changes in their biopsies, indicating that NK cell activation is rather associated with borderline and TCMR than with AMR rejection.

Regarding the mechanism for these phenotypic alterations, decreased CD16 surface expression was shown to occur in the course of *in vitro* stimulation. Treatment with CNI, but not with mTOR inhibitors, significantly suppressed both CD16 down-modulation and induction of cytokine secretion. IFN-γ production by NK cells of kidney transplant recipients was retained after stimulation with PMA/Ionomycin or with HLA-class-I negative K562 target cells, respectively. Degranulation and release of perforin and granzyme A/B were not impaired in KTx patients compared to healthy individuals. In contrast, induction of other cytokines like TNF-α, IL-13 or IL-31 by peripheral blood monocytic cells (PBMC) of kidney transpant (KTx) recipients was significantly reduced arguing for impaired NK and T cell-mediated cytokine responses due to immunosuppressive medication. These observations suggest that NK cells may be suitable as indicators for the individual immune status and as markers for an individualized adjustment of immunosuppression.

## Methods

### Patients and sample collection

The ethics committee of Hannover Medical School approved the collection of blood from healthy donors (MHH ethics committee No. 968–2011) as well as from kidney transplant recipients (KTR) (No. 5970.124). All participants have signed a written informed consent based on written patient information prior to sample collection. PBMC were prepared by Ficoll (Biochrom, Germany) separation and either cryopreserved or used directly. Details of patients (n = 29) and healthy donors (n = 11) are given in Table 1. PBMC of four randomly selected KTR were subjected to further *in vitro* analysis. The categorization of the KTx patients according to transplant pathology was based upon a histopathological examination of a (protocol or indication) transplant biopsy on the day of the sample collection according to the current Banff-classification [20]. Induction therapy using the IL-2 receptor antagonist basiliximab was given to 17 patients (58.6%), seven (24.1%) patients had no induction therapy, three (10.4%) were treated with ATG and two (6.9%) with rituximab. Standard immunosuppression comprised oral triple immunosuppression with cyclosporin A (CsA) or tacrolimus (Tac) in combination with corticosteroids and the nucleosid analogon mycophenolate mofetil (MMF), a subset of patients received Tac plus sirolimus (T/S).

**Table 1:** demographic characterization of the study cohort of kidney transplanted patents and healthy donors.

	parameter	Number (%)
**healthy donors (hd)**		11
**gender**	male	6 (54.5%)
	female	5 (45.5%)
**age**	median years (range)	35y (25–47y)
**kidney Tx patients**		29
**gender**	Male	17 (58.6%)
	Female	12 (41.4%)
**age**	median years (range)	41y (14–73y)
time between Tx and biopsy (Bx)	≤ 3 month	10 (34.5%)
	6 months	8 (27.6%)
	≥9 month	11 (38.0%)
**treatment**		
**induction therapy**	Basiliximab	17 (58.6%)
	ATG	3 (10.4%)
	Rituximab	2 (6.9%)
	No induction	7 (24.1%)
**immunosuppression**	Tac / MMF or MPA / prednisolon	16 (55.2%)
	mean Tac trough level	8.76 ± 2.99 ng/ml
	CsA / MMF or Azathioprin / predinisolon	8 (27.6%)
	mean CsA trough level	87.57 ± 46.73 ng/ml
	Tac/Sirolimus/Prednisolone	5 (17.2%)
	mean Tac trough level	8.75 ± 3.77 ng/ml
**co-medication**	MMF / MPA	23 (dosis 0,36–5 g)
	Prednisolone	27 (dosis 5–15 mg)
	Azathioprine	1 (75 mg)
**pathology of Bx** (day of blood sample)	Unsuspicious	13 (44.8%)
	AMR	6 (20.7%)
	TCMR	5 (17.2%)
	Borderline	4 (13.8%)
	Others (glomerulonephritis)	1 (3.5%)
**donor-specific antibodies (DSA)**	DSA-positive	9 (31.0%)
	DSA-negative	20 (69.0%)
**KTx patients (T cell assays)**		4
**Gender**	Male	3 (75%)
	Female	1 (25%)
**Age**	median years (range)	35.5 (14–68)
**Time between Tx and Bx**	6 months	1 (25%)
	≥9 months	3 (75%)
**treatment**	Tac / MMF or MPA / prednisolon	2 (50%)
	mean Tac trough level	6.5 ± 0.7 ng/ml
	CsA / MMF or Azathioprin / predinisolon	1 (25%)
	mean CsA trough level	30 ng/ml
	Sirolimus/Prednisolone	1 (25%)
**Co-medication**	MMF / MPA	3 (0.36–1.5 g)
	Prednisolone	4 (5–15 mg)
**pathology of Bx** (day of blood sample)	Unsuspicious	3(74%)
	AMR	1 (25%)
**donor-specific antibodies (DSA)**	DSA-positive	1 (25%)
	DSA-negative	3 (75%)

### NK cell isolation from PBMC

NK cells were MACS-isolated using a NK cell negative isolation kit (Miltenyi, Germany) according to the manufacturer’s instructions. Purity as determined by flow cytometry was >90% CD3^-^CD56^+^ NK cells.

### Characterization of lymphocyte subsets by flow cytometry

PBMC were incubated with directly labeled mAbs for 30 min at 4°C, washed with PBS containing 2.5% FBS (Life Technologies, NY,USA) and 0.1% sodium azide (Sigma-Aldrich, St. Louis, MO, USA) and fixed with PBS containing 1% paraformaldehyde (Merck, Germany). All Abs and conjugates are summarized in S1 Table.

### Intracellular cytokine and NFAT2 staining

For combined surface and intracellular staining, cells were incubated with mAbs staining surface molecules for 30 min at room temperature, washed with PBS, permeabilized with fixation/permeabilization buffer for another 30 min and incubated with mAbs staining intracellular NFAT2 and IFN-γ in permeabilization buffer (eBiosciences, San Diego, USA) for 30 min at 4°C. mAbs for cell surface staining are given in S1 Table, mAbs for intracellular staining: NFAT2-AF488 (7A6, Biolegend, San Diego, USA) and IFN-γ-PE (B27, Becton-Dickinson, USA). After two washings steps with PBS, cells were analyzed by flow cytometry (LSR II, Becton-Dickinson, Franklin Lakes, NJ, USA), data were processed using FACS-Diva software 6.1.2.

### Stimulation of PBMC with PMA/Ionomycin

PBMC or isolated NK cells (1 x10^6^/ml) were pre-incubated for 20 minutes with 5 μM inhibitor (Cyclosporin A, Tacrolimus, Sirolimus, Everolimus, myophenolic acid (MPA)) or solvent (DMSO or ethanol). Cells were stimulated for 6h or 24h with 50 ng/ml phorbol myristate acetate (PMA) and 2.5 μg/ml ionomycin (both Sigma-Aldrich) in RPMI1640 supplemented with 2mM L-glutamine, 1mM sodium pyruvate, 100U/ml penicillin/streptomycin containing 10% fetal bovine serum (FBS, TM) or left untreated. Cells and supernatants were harvested after 6h or 24h; cells were analyzed for surface and intracellular molecules, supernatants were analyzed by multiplex cytokine analyses.

### Quantification of cytokines by multiplex technology

Cytokine concentrations in supernatants of treated PMBC or isolated NK cells were quantified by the Luminex-based multiplex technique according to the manufacturer’s instructions (BioRad, Hercules, USA). Standard curves and concentrations were calculated with Bio-Plex Manager 6.1 software.

### Quantification of Interferon-γ secreting NK cells by ELISpot

The human IFN-γ ELISpot antibody pair (BD) was used for assays performed according to the manufacturer’s instructions. PBMC of KTR or healthy individuals were stimulated with K562 cells with a ratio of 3:1 in triplicates. Stimulation was performed for 18h in TM, IFN-γ spots were quantified by Immunospot S5 UV-Reader, Immunospot 5.1 software (CTL, Shaker Heights, OH, USA). Supernatants were collected and analyzed for perforin and granzyme A/B content using multiplex technology. To control for the individual proportion of NK cells, PBMC were simultaneously analyzed by FACS, percentages of NK cells were measured and IFN-γ ELISpots, perforin and granzyme A/B concentrations of all experiments were normalized to 10,000 NK cells per well.

### Statistical data analysis

Kolmogorov-Smirnov and d'Agostino & Pearson omnibus normality tests were performed to determine Gaussean distribution before application of Student’s t test or two-sided One-way-ANOVA with Tukey’s post-test or Dunnett’s Multiple Comparison test. For non-parametric data, either a Mann-Whitney-U-test or, for multiple comparisons, Kruskal-Wallis test with Dunn’s post-test were chosen. Significance was defined as p≤ 0.05 (GraphPadPrism 5.01, La Jolla, CA, USA).

## Results

### NK cells of kidney Tx recipients show reduced expression of CD16, CD226 and CD161 and increased expression of activation markers

**Fig 1:**
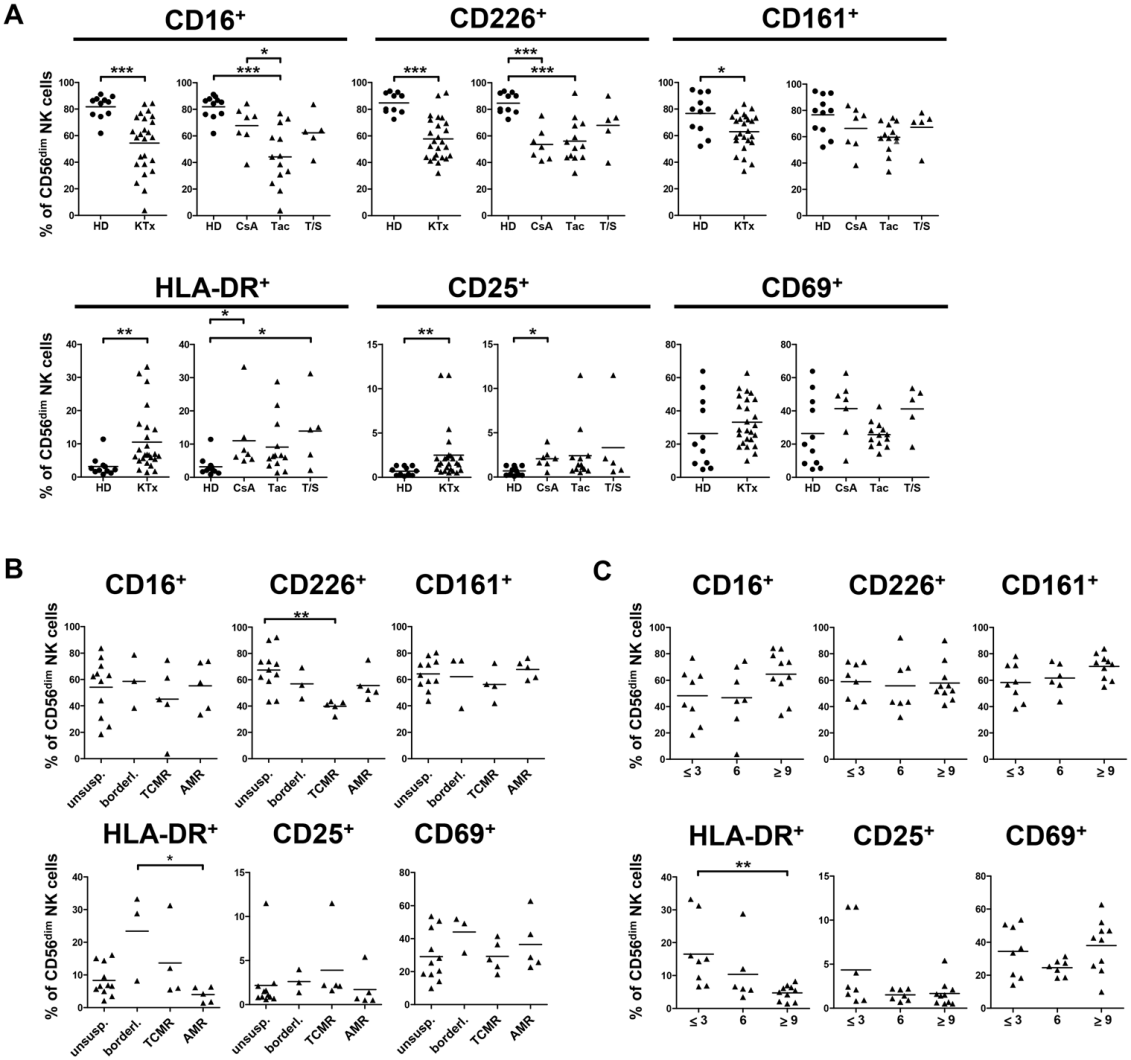
Surface expression of CD16, CD226 and CD161 is significantly reduced in KTx patients, while CD25, CD69 and HLA-DR surface expression is increased. Phenotypic characterization of peripheral NK cells from healthy individuals (n = 11, circles) and KTx patients (n = 29, triangles) was performed by flow cytometry. (A) CD16, CD226 (DNAM-1), CD161, HLA-DR, CD25 and CD69 expression was determined on CD56^dim^ NK cells, and compared between healthy donors (HD) and KTx patients (left plots). Displayed are mean values using unpaired Student’s t test (* = p≤0.05, ** = p≤0.01 and *** = p≤0.001, only significant values are shown). The impact of immunosuppression (right plots) was determined by grouping patients according to their immunosuppressive regimen: CsA, Tac or combination of Tac and Sir (T/S). Displayed are mean values, D'Agostino & Pearson omnibus normality test was performed to determine Gaussian distribution, subsequently either One-way-ANOVA or Kruskal-Wallis test were used to determine statistical significance. (B) Patients were grouped according to the histopathology of their biopsies (Banff classification): unsuspicious, borderline, T cell-mediated (TCMR) or antibody-mediated (AMR) rejection. (C) The impact of time after Tx was determined by grouping patients according to the time interval after Tx: ≤3, 6 or ≥ 9 months. Data are shown as scatter plots and display mean values. Asterisks indicate p-values * = p≤0.05, ** = p≤0.01 and *** = p≤0.001, only significant values are shown.

In our cohort of KTx recipients, we could previously demonstrate that their NK cell phenotype was altered compared to healthy donors with respect to subset composition, especially the CD56^dim^ NK subset [18]. These alterations correlated with the immunosuppressive treatment of KTx patients with CNI and/or mTORi. Here, additional activating and inhibitory receptors were analyzed in PBMC of a larger patient cohort (n = 29) and compared to healthy individuals (n = 11). In contrast to CD16 (FcγRIII), which is expressed by CD56^dim^ NK cells, CD226 and CD161 are expressed by both NK subsets. In KTx recipients, surface expression of these receptors was significantly reduced, in particular in the CD56^dim^ subset (Fig 1A, S1A–S1C Fig) while NKG2D expression was not affected. Receptor down-modulation on NK cells derived from KTx patients appeared to be coordinated because low CD16 density was accompanied by low CD226 (p = 0.016) and CD161 (p = 0.004) expression (S1D Fig, S2 Table). This modified NK cell repertoire was associated with an activated phenotype, since both CD56^dim^ and CD56^bright^ NK cells of KTx patients showed increased expression of three activation markers, i.e. human leukocyte antigen (HLA)-DR, CD25 and CD69. When patients were grouped according to their immunosuppressive regimen, CD56^dim^ NK cells of Tac-treated patients showed stronger reduction of CD16, CD226 and CD161 expression compared to patients treated with CsA or Tac plus sirolimus (Tac/Sir, T/S) reaching statistical significance only for CD16 between CsA- and Tac-treated patients. The HLA-DR^+^, CD25^+^, CD69^+^ activated phenotype was more pronounced in Tac-treated patients without reaching statistical significance (Fig 1A, S1C Fig).

With respect to inhibitory receptors, NK cell subsets defined by KIR and CD94/NKG2A expression were compared between healthy donors and KTx patients. KIR expression is generally restricted to the CD56^dim^ NK cell subset and characterizes potentially alloreactive NK cells based on KIR ligand mismatch between donor and recipient [21]. In KTx patients, the proportion of all KIR^+^CD56^dim^ NK cell subsets was reduced reaching statistical significance for GL183^+^ NK cells expressing the inhibitory KIR2DL2/3 or the activating KIR2DS2 receptors and multiple KIR^+^ NK cells (S2A Fig). The CD94/NKG2A heterodimer can be detected on virtually all NK cells but at different densities ranging from highest expression on CD56^bright^ NK cells via intermediate to low expression on CD56^dim^ NK cells which is associated with maturation [22–24]. The proportion of CD94^+^NKG2A^+^ CD56^bright^ NK cells was also significantly increased in KTx patients compared to healthy individuals (S2B Fig).

When patients were grouped according to histopathology of their biopsies, i.e. classified as unsuspicious biopsy or rejection with borderline, TCMR or AMR changes according to the Banff criteria, alterations in NK cell phenotype could be associated with histopathology. CD226 expression on CD56^dim^ NK cells was significantly lower in the TCMR group than in patients with unsuspicious, borderline or AMR biopsies while CD16 and CD161 did not significantly differ between these groups (Fig 1B, S3A Fig). Increased HLA-DR expression was observed on CD56^dim^ NK cells of patients with rejection reaching statistical significance between borderline and AMR patients. When grouped according to the time interval after Tx, no significant differences in CD16, CD161 and CD226 expression were observed but NK cells of patients transplanted longer than 9 months displayed significantly lower HLA-DR expression on CD56^dim^ NK cells compared to patients after < three months after Tx (Fig 1C, S3B Fig). Decreased HLA-DR expression was associated with higher CD16 expression at later phases after Tx, though not reaching statistical significance. Since there was no correlation with time after Tx for the other receptors, the time-dependent modulation of CD16 and HLA-DR argues for their special sensitivity to the onset of intense immunosuppression in the first three to six months after Tx. Thus, CD16 and HLA-DR may serve as suitable markers for the adjustment of early NK cell activation *vs*. immunosuppression.

### NK cells respond to *in vitro* stimulation with CD16 down-regulation and IFN-γ production which is suppressed by CNI but not by mTOR inhibitors

**Fig 2:**
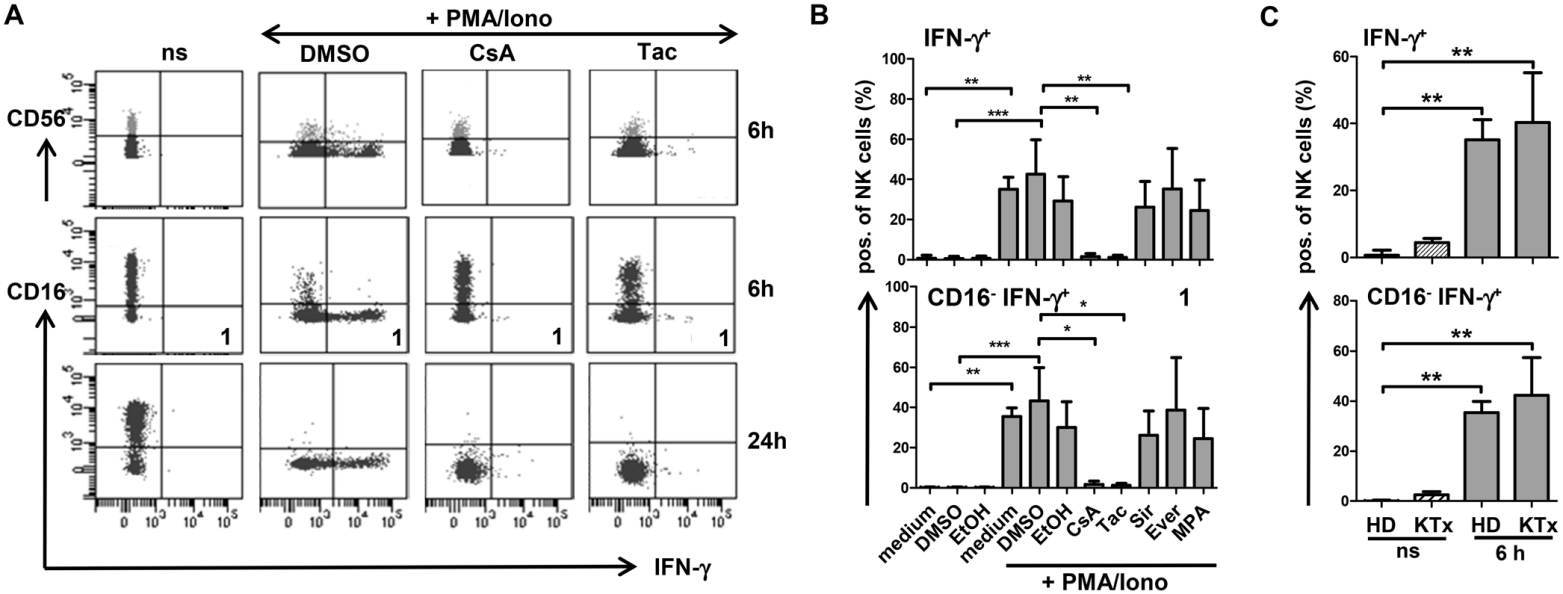
CD16 down-regulation is associated with IFN-γ induction following stimulation of NK cells from healthy individuals and KTx patients. PBMC of healthy donors (n = 6) or KTx-patients (n = 4) were pre-incubated with 5 μM inhibitor or equal concentrations of DMSO solvent, stimulated with P/I for 6 or 24 h, respectively and stained for surface CD3, CD56, CD16 and intracellular IFN-γ. (A) FACS dot plot analysis of gated CD3^-^CD56^+^ NK cells of one representative healthy donor is shown. CD16^-^IFN- γ^+^ subset used for statistical evaluation was labeled as 1. (B) Corresponding statistics of NK cells of 6 healthy individuals regarding IFN-γ-positive subsets in combination with CD16 after 6h stimulation are shown as mean values ± standard deviation compared by Kruskal-Wallis test followed by Dunn’s Multiple Comparison test (* = p<0.05, ** = p<0.01, *** = p<0.001, only significant values are shown). (C) Corresponding statistics of NK cell subsets of healthy donors (n = 6) in comparison with KTx patients (n = 4) after 6h stimulation are displayed.

In order to investigate the molecular mechanism of decreased CD16 expression in CD56^dim^ NK cells of KTx patients, PBMC of healthy donors were stimulated *in vitro* in the presence or absence of immunosuppressive drugs, i.e. CsA, Tac, Sir, Ever or MPA. After 6h stimulation with PMA/ionomycin (P/I), CD16 was significantly down-regulated and, thus, the majority of CD56^dim^ NK cells became CD16-negative (Fig 2A and 2B). Incubation with inhibitors alone had no effect on CD16 expression. Intracellular staining revealed that IFN-γ induction was associated with loss of CD16 expression (panel 1) since virtually no IFN-γ/CD16-double-positive NK cells were detected and remaining CD16-positive NK cells did not produce IFN-γ. In the presence of CsA or Tac, CD16 down-regulation was impaired while mTORi and MPA had minor effects. Upon CNI treatment, IFN-γ production was blocked almost completely, whereas treatment with mTORi or MPA could not significantly inhibit IFN-γ production. While the inhibitory effect of CNI on IFN-γ production was still continued after 24h, a block of CD16 down-regulation could no longer be observed (Fig 2, S4A Fig). These *in vitro* findings indicated that both IFN-γ production which depends on transcriptional activity *via* NFAT signaling and CD16 down-regulation were both suppressed by CNI but not by mTORi and MPA.

Since we observed reduced proportions of KIR^+^ CD56^dim^ and increased CD94^+^NKG2A^+^ NK cells in KTx patients compared to healthy individuals (S2A and S2B Fig), expression of KIR and CD94/NKG2A was analyzed *in vitro* in healthy donor NK cells under stimulatory and inhibitory conditions. Neither CNI nor mTORi had an impact on KIR expression on CD56^dim^ NK cells but CD94 expression was modulated upon P/I stimulation by shifting from a CD94^dim^ to a CD94^bright^ phenotype (S4B and S4C Fig). NKG2A did not increase simultaneously and, thus, CD94 single-positive NK cells were enriched.

### NK cells of KTx recipients under immunosuppression retain their ability to respond to stimulation with P/I or K562 target cells

**Fig 3:**
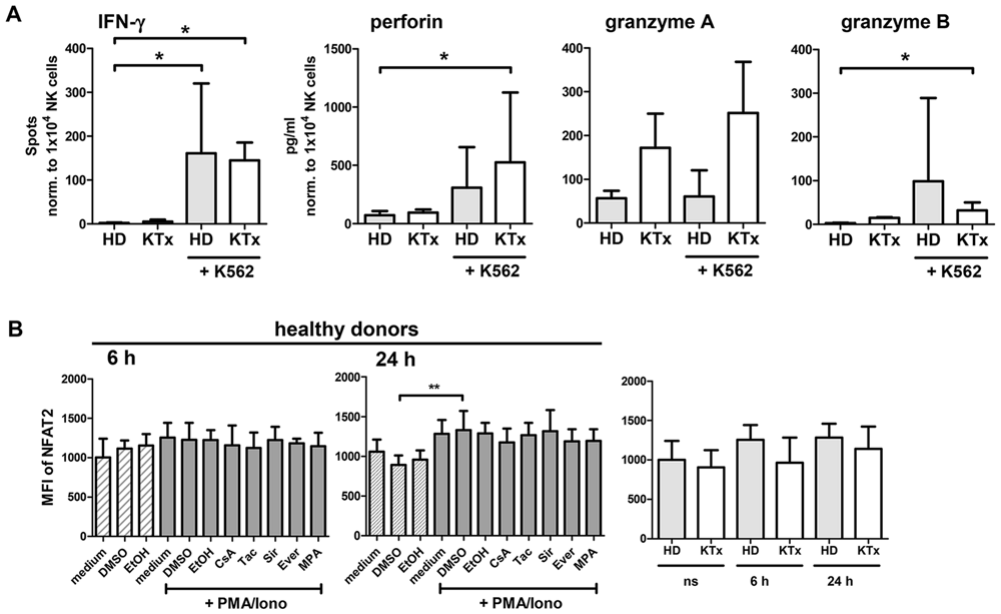
NK cell activity in response to K562 target cells as well as intracellular NFAT2 expression is retained in KTx patients compared to healthy individuals. (A) PBMC of healthy donors (n = 4, grey bar) or of KTx patients (n = 4, white bar) were incubated for 18h with K562 target cells and activation was quantified by IFN-γ ELISpot and multiplex analyses of supernatants for perforin and granzyme A/B. For statistical analyses, spots were normalized to 10.000 NK cells per well and mean values ± standard deviations are depicted, compared by Kruskal-Wallis test followed by Dunn’s Multiple Comparison test. (B) PBMC of healthy donors (n = 6) were pre-incubated with immunosuppressive drugs (5 μM) or DMSO solvent for 20 min and either left unstimulated (shaded bars) or P/I stimulated for additional 6h or 24h, respectively (grey bars, left and middle graph). KTx recipient-derived PBMC (n = 4) were stimulated identically, cells were stained intracellular for total NFAT2 and analyzed by flow cytometry. The right plot shows total NFAT2 in healthy donors compared to KTx patients after 24h stimulation. Mean values and standard deviations are displayed compared by two-sided One-way-ANOVA test (* = p≤0.05, ** = p≤0.01, *** = p≤0.001, only significant values are shown).

Since the NK cell repertoire of KTx patients under immunosuppression showed remarkable alterations, we investigated if this particular phenotype was associated with impaired NK cell function. Upon stimulation with P/I for 6h or 24h, CD16 expression on CD56^dim^ NK cells was down-regulated at the same level in patients as in healthy donors and equivalent amounts of IFN-γ were produced (Fig 2C, S4D Fig). To test whether NK cells from KTx patients remained responsive to stimulation with HLA class I-negative K562 target cells, IFN-γ ELISpot assays were performed. In order to control for individual proportions of NK cells in healthy donors and KTx recipients, IFN-γ spots were normalized to 10,000 NK cells (Fig 3A). No significant differences in IFN-γ-secreting NK cells in response to K562 cells were observed between healthy individuals and KTx recipients. The supernatants harvested from these K562 stimulations were used to quantify perforin and granzyme A/B secretion and, again, no significant differences were measured between patients and healthy donors. Thus, NK cells of KTx recipients seem to retain their capacity to respond to strong, non-specific stimulation such as P/I as well as to specific stimulation by K562.

### NFAT2 is induced in NK cells of KTx recipients and healthy donors at comparable levels upon P/I stimulation

Since we identified CNI to be most effective in blocking NK cell functions, we wanted to investigate the signaling pathway targeted by these drugs, i.e. the calcineurin-NFAT pathway by focusing on NFAT2. NFAT2 is dephosphorylated by calcineurin upon stimulation and mediates sustained signal transduction and transcriptional activation by its inducible isoform [16]. NFAT2 was detected by intracellular staining [25] in resting NK cells of healthy donors without significant modulation after 6h P/I stimulation but with a significant increase after 24h stimulation which was not affected by CNI treatment (Fig 3B). In KTx patients, NFAT2 basal expression and kinetics were comparable to healthy donors. Thus, the changes in NK cell phenotype and function in KTx recipients under immunosuppressive medication were not directly caused by altered intracellular NFAT2 levels.

### CNI impair cytokine production of PBMC and isolated NK cells *in vitro* more efficiently than mTOR inhibitors

**Fig 4:**
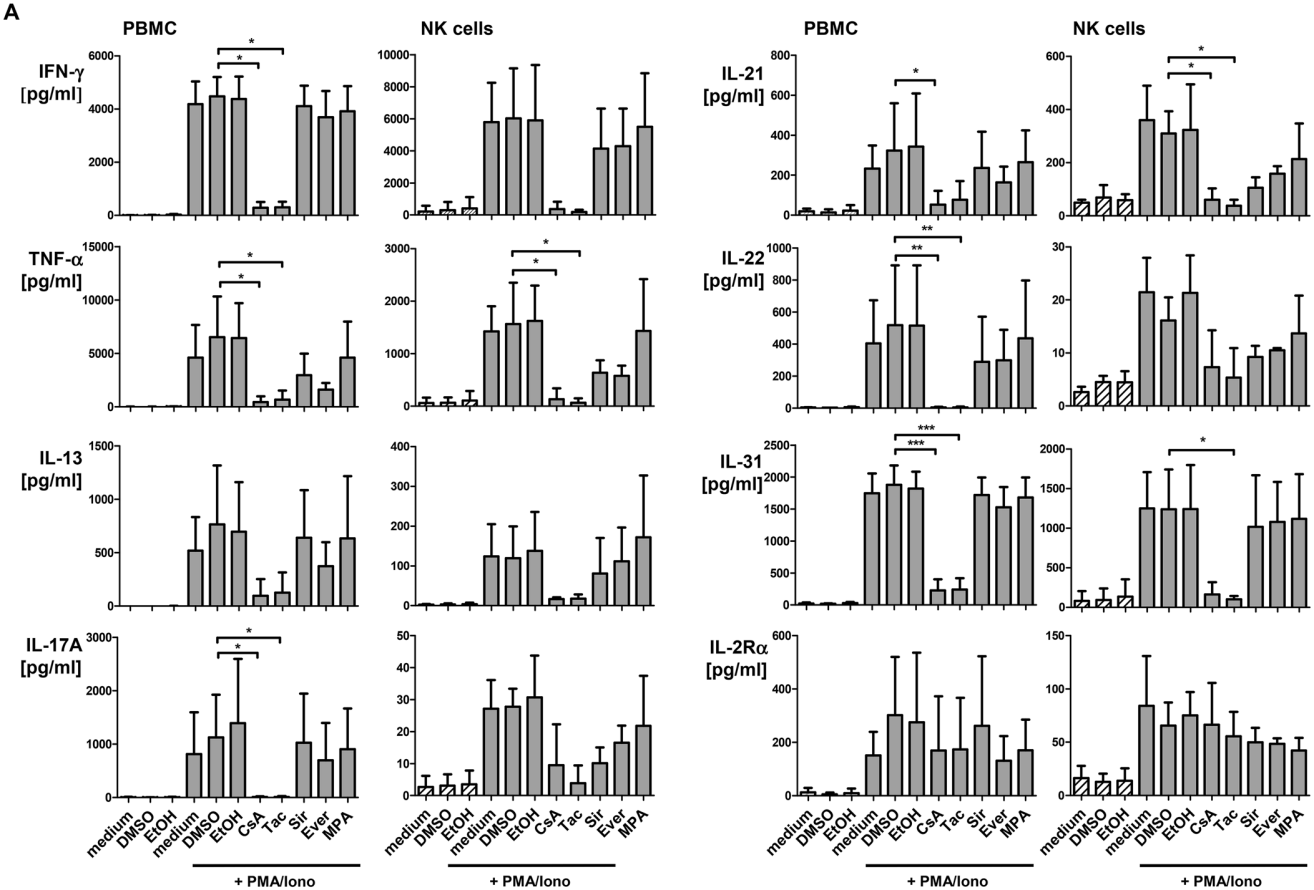
CNI but not mTORi suppress cytokine production of PBMC and isolated NK cells of healthy donors. PBMC of healthy donors (n = 6) were pre-incubated for 20 min with 5 μM inhibitor or DMSO solvent, stimulated with P/I for 24h, supernatants were collected and analyzed for cytokine secretion. Mean values ± standard deviation are shown. To determine statistical significance, Kruskal-Wallis test with Dunn’s post test comparing the different inhibitor treatments to DMSO control was performed. NK cells were negatively MACS-isolated from healthy donor PBMC and stimulated as described. To determine statistical significance, One-Way-ANOVA with Dunnett’s multiple comparison test was performed (* = p≤0.05, ** = p≤0.01, *** = p≤0.001, only significant values are shown).

In order to confirm our observations of intracellular IFN-γ production with other cytokines, supernatants of PBMC and isolated NK cells following 24h P/I stimulation in presence or absence of CNI, mTORi or MPA were harvested and applied to the multiplex protein technology to determine the Th1/Th2/Th17/Th22 profile. In PBMC, secretion of IFN-γ, TNF-α, IL-17A, IL-21, IL-22 and IL-31 was significantly suppressed by both CNI, while mTORi as well as MPA exerted only minor inhibitory effects without statistical significance (Fig 4A). A similar cytokine profile was detected for isolated NK cells, however, CNI-mediated suppression reached statistical significance only for TNF-α, IL-22 and IL-31. NK cells primarily produced IFN-γ, TNF-α, IL-13, IL-21 and IL-31 after P/I stimulation, lower amounts of IL-17A and IL-22 and hardly contributed to IL-4, IL-10, IL-17F production (S5A Fig). Of note, the capacity to produce the Th2 cytokine IL-31 has not been described for NK cells so far [26]. The efficacy of CNI in suppression of these cytokines argues for a rather broad spectrum of NFAT-dependent target genes across Th1/Th2/Th17 cytokine families. In contrast to cytokines, secretion of soluble IL-2Rα (sCD25) was neither suppressed by CNI nor by mTORi or MPA suggesting that cellular processes that are independent from transcriptional regulation *via* NFAT such as shedding of IL-2Rα or degranulation of cytotoxins, for instance, cannot be inhibited by these immunosuppressive drugs.

### PBMC of KTx patients show retained capacity to produce IFN-γ while secretion of other cytokines is significantly impaired

**Fig 5:**
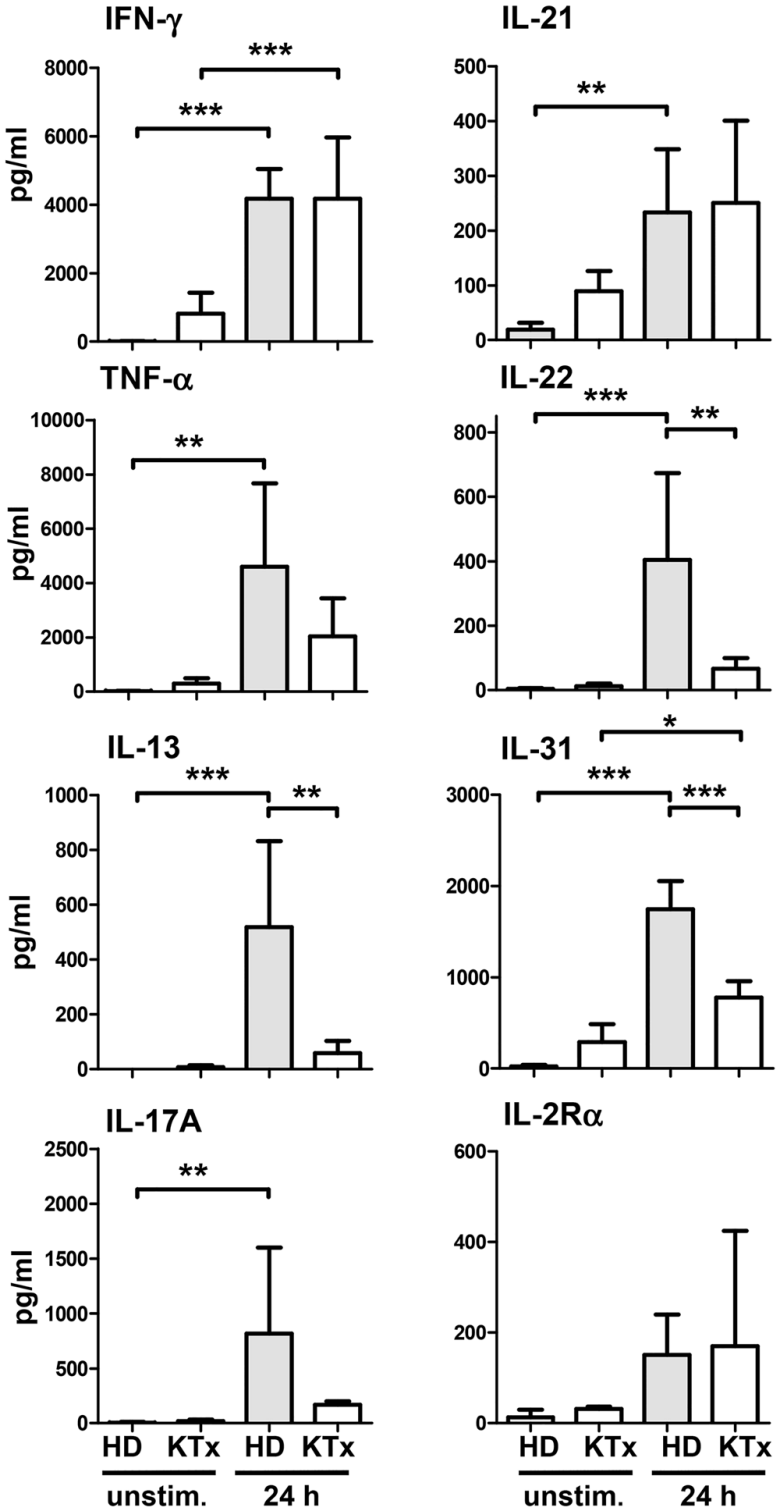
Cytokine response of PBMC derived from KTx patients is partially impaired compared to healthy individuals. PBMC of KTx patients (n = 4, white bars) were stimulated for 24h with P/I or left untreated as described, supernatants were collected, analyzed for cytokine production and compared to P/I stimulated PBMCs of healthy donors (n = 6, grey bars). Data are represented as mean values compared by two-sided One-way ANOVA test with Tukey’s post test (* = p≤0.05, ** = p≤0.01, *** = p≤0.001, only significant values are shown).

The multiplex analyses of supernatants from P/I stimulated PBMC of KTx recipients confirmed our observations of intracellular IFN-γ production (Fig 2C). Unstimulated PBMC of KTx patients showed slightly, though not significantly, elevated cytokine levels. The capacity to respond to P/I stimulation was selectively retained for certain cytokines (Fig 5): while induction of IFN-γ and IL-21 secretion as well as shedding of IL-2Rα was comparable, secretion of other cytokines was reduced in KTx patients compared to healthy individuals. Significantly lower concentrations were detected for IL-13, IL-22, IL-31 (Fig 5) and IL-4 (S5B Fig). Though not reaching statistical significance, secretion of TNF-α, IL-17A, IL-10 and IL-17F was impaired in KTx recipients suggesting that immunosuppressive treatment differentially affects cytokine responses and may, thus, impinge on the Th1/Th2/Th17 balance.

## Discussion

Several studies underline the role of NK cells in kidney transplantation. An involvement of NK cells in the molecular landscape of kidney biopsies with AMR pathology has recently been reported [27]. In a murine kidney transplant model based on hybrid resistance, NK cells were shown to mediate long-term allograft injury in the absence of T and B cells [28]. In this context of missing-self recognition, an influence of certain KIR genes was proposed as KIR2DS3 without cognate HLA ligand was associated with better, with ligand with worse effects on kidney function [13]. In order to improve our understanding of the NK cell repertoire, function and the influence of immunosuppression in our patient cohort after KTx, we investigated receptor expression and response to *in vitro* stimulation of NK cells derived from KTx recipients compared to healthy individuals. Peripheral NK cells derived from patients displayed a significantly altered phenotype characterized by down-regulation of several NK cell receptors and elevated expression of activation markers. Compared to healthy individuals, CD16, CD161 and CD226 surface expression was significantly decreased on NK cells of KTx patients and the KIR^+^ NK cell subset was substantially reduced. In contrast to NK receptors, expression of activation markers like CD69, CD25 and HLA-DR was significantly elevated in KTx patients indicating that this altered phenotype may result from constitutive activation. Down-modulation of CD226 was even associated with pathological staging of kidney biopsies whereby a significantly reduced CD226 expression was detected on NK cells from patients with TCMR. This association implies an involvement of CD226 in NK cell recognition of the allograft, analogously to the contribution of CD226 in recognition of melanoma cells [29]. Furthermore, HLA-DR and CD16 expression normalize with time after Tx which could reflect declining activation over time. Thus, these three markers may be informative regarding long-term adjustment of immunosuppression and development of rejection, AMR, in particular. Thus, these markers represent candidates for an evaluation in studies addressing the role of NK cells in solid organ transplantation.

In a recent study, DSA- and non-DSA-HLA-antibody-positive kidney transplant patients were reported to display elevated CD56^bright^ and NKG2A^+^ NK cells compared to the HLA-antibody-negative group. In our cohort, we also observed increased expression of the inhibitory heterodimer CD94/NKG2A on CD56^bright^ NK cells. Upon *in vitro* stimulation, CD94 was induced without simultaneous NKG2A induction. In a study of Wang et al., a decrease in KIR^+^ and CD16^+^ NK cells within the CD56^dim^ NK cell fraction was reported upon stimulation with cytokines or target cells in the presence of CsA for several days [30]. In their experimental setting, the effect resulted from reduced proliferation of CD56^dim^ NK cells and a reciprocal increase in CD16^-^ NK cells and their cytokine production after >7 days. In contrast, in our short-term setting of P/I stimulation and simultaneous exposure to CsA, Tac, mTORi or MPA, loss of CD16 was already achieved 6h after stimulation and associated with the induction of cytokine expression dependent on calcineurin activity. Remarkably, significant differences between our CsA- and Tac-treated KTx patients were identified with respect to their capacity to control down-regulation of activating NK receptors like CD16 and CD6 [18]. These differences may relate to the different binding capacity of the Tac-FKBP12 and the CsA-cyclophilin complexes to the phosphatase calcineurin [31] as well as to the different effectiveness of the co-medication. Both inhibitor/ immunophilin-complexes block the active center of the Ser/Thr phosphatase which prevents from subsequent NFAT dephosphorylation and translocation into the nucleus and inhibits transcription of downstream targets like IL-2, IFN-γ or GM-CSF. The observation that upon *in vitro* stimulation of NK cells from healthy individuals, no significant differences were seen between CsA and Tac treatment argues for systemic long-term effects of individual immunosuppressive drug regimens on the immune system of kidney recipients and the relevance of co-medication [19]. In contrast to CNI, mTORi are known to block the PI3K/Akt pathway and impinge on costimulatory signal transduction in T cells but also on general protein translation in immune and non-immune cells. Surprisingly, mTOR inhibitors displayed little inhibitory efficacy regarding receptor modulation and cytokine production which may be clinically relevant in terms of the risk of underimmunosuppression with this class of drugs.

The variety of NK cell-related differences between KTx recipients and healthy individuals argues for a cooperation of different mechanisms. Reduced CD16 expression on NK cells in KTx patients could be identified as activation-induced down-regulation by signals involving calcium and PKCϑ signaling. Following P/I stimulation, loss of CD16 surface expression was accompanied by the induction of IFN-γ expression strongly arguing for a direct link between activating signal pathways and receptor down-modulation. Since in our study, primarily CD56^dim^ NK cells produce IFN-γ, we could exclude that these findings were biased by cytokine production of CD16^-^ CD56^bright^ NK cells independently from their activation status. Activation-induced CD16 modulation was reported earlier in response to IL-12 + IL-18, K562 and receptor crosslinking of CD226, NKp46 and NKG2D [32]. In contrast to IL-12 + IL-18, IL-2 stimulation alone was unable to mediate CD16 down-regulation (S6 Fig) which underlines the dependence on Ca^++^ and PKCϑ signaling. Decreased CD226 expression in NK cells was reported after co-culture with target cells expressing its ligands CD155 and CD166, indicating that this receptor regulation was induced by chronic ligand exposure [33]. Thus, the altered NK cell phenotype observed in KTR is likely to result from sustained activation following transplantation.

Our results demonstrate that short-term P/I stimulation as well as immunosuppression do not directly influence KIR expression although we observed generally reduced fractions of KIR^+^ NK cells in peripheral blood of kidney transplant recipients. Again, this argues for a rather long-term process of KIR modulation in these patients. Meehan et al. observed a decline in KIR expression in lung transplant recipients and attributed this finding to replacement of differentiated NK cells in the peripheral blood by a more immature precursor NK cell population [34]. This interpretation was supported by our finding of simultaneously increased fractions of CD94/NKG2A^+^ CD56^bright^ NK cells in the blood of KTx patients. The concept of constitutive NK cell activation was strengthened further by significantly increased expression of activation markers as CD69, HLA-DR and CD25 in KTx patients and by the finding that unstimulated PBMC of KTx recipients displayed higher, though not significantly, background levels of certain cytokines like IFN-γ, IL-21 and IL-31.

Regarding the functional capacity, NK cells of KTx recipients under immunosuppression retained their reactivity to P/I stimulation because IFN-γ production, CD16 down-regulation and NFAT2 regulation were not significantly reduced compared to healthy individuals. Likewise, NK cells retained their capacity to respond to stimulation with K562 target cells with IFN-γ production and degranulation. Upon P/I stimulation, release of certain NFAT-dependent cytokines as IL-13, IL-22, IL-31 and IL-4 by PBMC of KTx patients was significantly lower than in healthy individuals. A similar, non-significant trend was observed for TNF-α,IL-17A/F and IL-10, whereas the capacity to produce IFN-γ, IL-21, cytotoxins and sCD25 was retained which argues for a differential regulation of cytokines and cytotoxins and context-dependent long-term effects of the immunosuppressive medication on the recipient immune status. Future studies will have to show, whether different histopathologies translate into differentially well preserved immune functions. Our analyses further demonstrate that the functional restrictions in terms of cytokine production do not apply to NK cell cytotoxicity. Reports regarding the *in vitro* effect of CNI on NK cell degranulation are controversial since they propose reduced [35], unaffected or even increased degranulation after CNI treatment depending on the respective experimental design [30]. Our own data of *in vitro* treatment with immunosuppressive drugs did not reveal a general impairment of lytic granule release in healthy donors, although there are individuals responding with reduced perforin and granzyme A/B release after target cell contact. Since degranulation does not require transcriptional activity induced by NFAT, the CNI effect on degranulation may rather depend on the mode of stimulation and its intracellular wiring of signals [35].

In our study, we could confirm that NK cells do not differ generally from T cells in their susceptibility to immunosuppression. While CNI prevented cytokine secretion of PBMC and isolated NK cells upon *in vitro* stimulation, mTORi had only minor effects. NFAT induction and nuclear translocation was reported as crucial step in NK cell activation [30,36]. Our *in vitro* assays showed that NK cells are not refractory towards immunosuppressive treatment and that NFAT2 is expressed in an activation-dependent manner. Furthermore, our results demonstrate that besides TNF-α and IFN-γ, NK cells may also produce substantial amounts of IL-31, IL-21 and IL-13 and less IL-17A, which extends their role in the coordination of Th1 *vs*. Th2 and Th17 immune responses. Altogether, we provide evidence that the altered NK cell phenotype in KTx patients results from their activation resulting from an underlying inflammatory process that needs to be controlled by immunosuppression. In this context, a recent review points to an non-specific activation of the innate immune system by so called “DAMPs” (damage-associated molecular patterns) that can be induced by stress situations of the renal graft including ischemia/ reperfusion damage [37]. These DAMPs, including reactive oxygen species, heat shock proteins and heparin sulfate, are recognized by pattern recognition receptors present at the cell surface such as toll-like receptors (TLR) and C-type lectin receptors expressed by innate immune cells triggering their activation [38]. Ligands from damaged cells within the transplant may then engage pattern recognition receptors, for example, the Toll-like receptors (TLRs) expressed by donor-derived antigen-presenting cells present within the allograft. On the basis of these various mechanisms, the differences between CsA- and Tac-treated patients and the association with pathological staging of biopsies may qualify NK cells as promising candidates for the establishment of peripheral biomarkers that may enable an individualized adjustment of immunosuppression and an early detection of clinically relevant rejection after solid organ transplantation.

## Supporting Information

S1 Fig FACS gating strategy and association between low CD16 density and low CD226 and CD161 expression. (A) Gating strategy for NK cells: NK cells where defined as CD3^-^CD56^+^ lymphocytes, the two major NK cell subsets where defined by intensity of CD56 expression (CD56^+++(bright)^ in box A, CD56^dim^ in box B). Figs 1 and 2 show the CD56^dim^ NK cell subset in combination with the respective markers, for example CD16 in box C. (B) FACS dot plot analyses for CD16 vs. CD56 expression of one representative healthy donor (left) and one representative KTx patient (right). (C) Phenotypic characterization of peripheral NK cells from healthy individuals (n = 11, circles) and KTx patients (n = 29, triangles) was performed by flow cytometry. CD16, CD226 (DNAM-1), CD161, HLA-DR, CD25 and CD69 expression was determined on all CD3^-^CD56^+^ and CD56^bright^ NK cells and compared between healthy donors (HD) and KTx patients (left plots). Mean values are displayed and compared by unpaired Student’s t test (* = p≤0.05, ** = p≤0.01 and *** = p≤0.001). The impact of immunosuppression in patients (right plots) was determined by grouping patients according to their immunosuppressive regimen: CsA, Tac or combination of Tac and Sir (T/S). Mean values are displayed, D'Agostino & Pearson omnibus normality test was performed to determine Gaussian distribution and subsequently either One-way-ANOVA or Kruskal-Wallis test was used to determine statistical significance. (D) Displayed is the correlation of CD226, CD161 or CD69 expression with CD16 expression levels on CD56^dim^ NK cells. For CD25, this correlation is also shown for CD56^+++^ NK cells. Statistical regression analyses are summarized in S2 Table.
(TIF)

S2 Fig KTx patients have significant less KIR double-positive NK cells compared to healthy donors. NK cells in PBMC of healthy donors (n = 11, circles) or KTx patients (n = 29, triangles) were analyzed by flow cytometry. (A) Surface expression of KIR2DL1 and 2DS1, KIR2DL2/3 and 2DS2/3 and KIR3DL1 and 3DS1 on all NK cells, CD56^dim^ and CD56^bright^ NK cells as well as the proportion of multiple KIR-positive NK cells was analyzed in HD and compared to KTx patients. Mean values are displayed, D'Agostino & Pearson omnibus normality test was performed to determine Gaussian distribution and subsequently either unpaired, two-sided t test or Mann-Whitney-U test was performed. (B) CD94, NKG2A and CD94/NKG2A surface expression on NK cells of healthy donors and KTR was measured as in C. Data are shown as scatterplots, mean values are displayed. Asterisks indicate the p-values, statistical significance was determined as described in C (* = p≤0.05, ** = p≤0.01 and *** = p≤0.001, only significant values are shown).
(TIF)

S3 Fig Pathological staging and time after Tx influences NK cell phenotype. (A) Patients were grouped according to the histopathology of their biopsies: unsuspicious, borderline, TCMR or AMR rejection. Statistical analyses were performed as described for Fig 1B. (B) The impact of time after Tx was determined by grouping patients according to the time interval after Tx: ≤3, 6 or ≥ 9 months. Data are shown as scatter plots and display mean values. Asterisks indicate p-values (* = p≤0.05, ** = p≤0.01 and *** = p≤0.001, only significant values are shown).
(TIF)

S4 Fig The inhibitory effect of CNI on CD16 down-regulation is lost after 24h stimulation, CD94 is induced and expression of KIR is not affected by immunosuppression *in vitro*. **(A)** Healthy donor PBMC (n = 6) were pre-incubated with 5μM inhibitor and left either unstimulated (shaded bars) or stimulated with P/I 24h (grey bars). Cells were stained for surface CD3, CD56, CD16 and intracellular IFN-γ. Statistics were performed using Kruskal-Wallis test with Dunn‘s post test (* = p<0.05, ** = p<0.01, *** = p<0.001, only significant values are shown), mean values ± standard deviation are displayed. (B) PBMC from healthy donors (n = 3) were pre-incubated with immunosuppressive drugs or control solvent and stimulated with P/I for 24h or left untreated. NK cells were analyzed for CD94, NKG2A expression by flow cytometry. (C) Statistics were performed for CD94, NKG2A, KIR2DL1/2DS1, KIR2DL2/2DS2, KIR3DL1/2DS1 positive NK cells within the respective subsets using One-way-ANOVA followed by Tukey‘s post test, mean values ± standard deviation are shown (* = p≤0.05, ** = p≤0.01, only significant values are shown). (D) FACS histogram and dot plot analyses are shown for intracellular IFN-γ and surface CD16 expression of gated CD3^-^CD56^+^ NK cells either unstimulated or stimulated with P/I for 6h or 24h of one representative KTx patient (ns = non-stimulated).
(TIF)

S5 Fig Cytokine production of PBMC and NK cells of healthy donors is inhibited by CNI treatment *in vitro* and can be impaired in KTx recipients in vivo. (A) PBMC of healthy donors (n = 6) were pre-incubated for 20 min with 5 μM inhibitor or DMSO solvent, stimulated with P/I for 24h, supernatants were collected and analyzed for IL-4, IL-10 and IL-17F secretion. Mean values ± standard deviation are shown. To determine statistical significance, Kruskal-Wallis test with Dunn’s post test comparing the different inhibitor treatments to DMSO control was performed. NK cells were negatively MACS-isolated from healthy donor PBMC and stimulated as described. To determine statistical significance, One-Way-ANOVA with Dunnett’s Multiple Comparison test was performed (* = p≤0.05, ** = p≤0.01, *** = p≤0.001, only significant values are shown). (B) PBMC of KTx patients (n = 4, white bars) were stimulated for 24h with P/I or left untreated as described, supernatants were collected, tested for IL-4, IL-10 and IL-17F secretion and compared to P/I stimulated PBMCs of healthy donors (n = 6, grey bars). Data are displayed as mean values compared by a two-sided One-way ANOVA with Tukey’s post test (* = p≤0.05, ** = p≤0.01, *** = p≤0.001, only significant values are shown).
(TIF)

S6 Fig CD16 down-regulation cannot be observed after IL-2 stimulation. (A) PBMC of healthy donors (n = 4) were incubated for 96h in the presence (grey bars) or absence (shaded bars) of IL-2 and CNI and mTORi treatment, respectively (inhibitor concentration: 10μM). NK cells were stained for CD56 and CD16 and analyzed by flow cytometry. CD16 expression of NK cells treated with CsA alone without addition of IL-2 could not be measured due to toxic effects of this drug on isolated NK cells without IL-2 supplementation.
(TIF)

S1 Table mAbs used for cell surface staining. (DOCX)

S2 Table statistical evaluation of surface receptor modulation (linear regression). (DOCX)
