# Supplementary figures and images for: NK Cells of Kidney Transplant Recipients Display an Activated Phenotype that Is Influenced by Immunosuppression and Pathological Staging
Source: PLoS One. 2015 Jul 6;10(7):e0132484. doi: 10.1371/journal.pone.0132484 (PMC4492590; doi:10.1371/journal.pone.0132484)

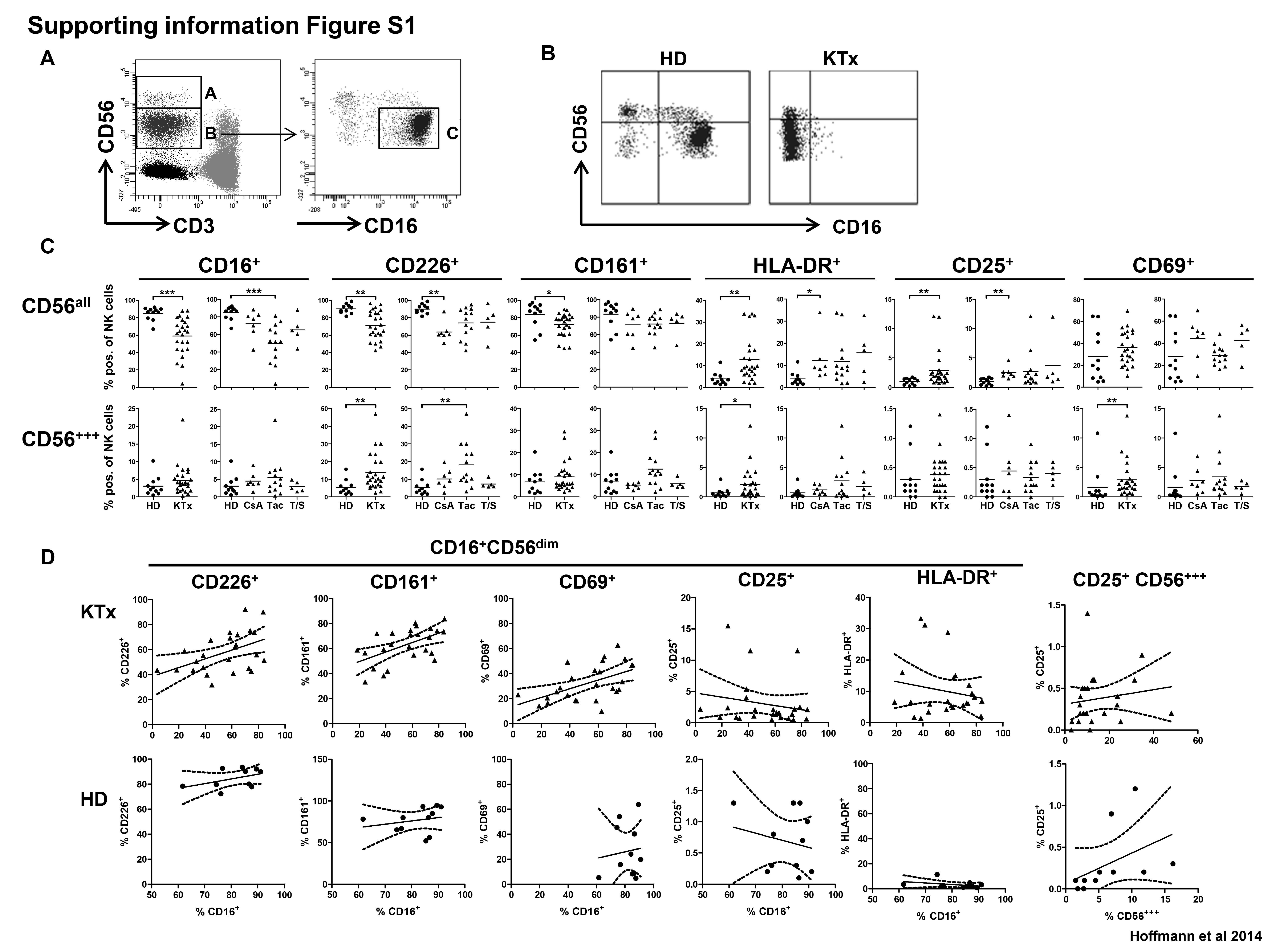

Supplement: S1 Fig — (A) Gating strategy for NK cells: NK cells where defined as CD3-CD56+ lymphocytes, the two major NK cell subsets where defined by intensity of CD56 expression (CD56+++(bright) in box A, CD56dim in box B). Figs 1 and 2 show the CD56dim NK cell subset in combination with the respective markers, for example CD16 in box C. (B) FACS dot plot analyses for CD16 vs. CD56 expression of one representative healthy donor (left) and one representative KTx patient (right). (C) Phenotypic characterization of peripheral NK cells from healthy individuals (n = 11, circles) and KTx patients (n = 29, triangles) was performed by flow cytometry. CD16, CD226 (DNAM-1), CD161, HLA-DR, CD25 and CD69 expression was determined on all CD3-CD56+ and CD56bright NK cells and compared between healthy donors (HD) and KTx patients (left plots). Mean values are displayed and compared by unpaired Student’s t test (* = p≤0.05, ** = p≤0.01 and *** = p≤0.001). The impact of immunosuppression in patients (right plots) was determined by grouping patients according to their immunosuppressive regimen: CsA, Tac or combination of Tac and Sir (T/S). Mean values are displayed, D'Agostino & Pearson omnibus normality test was performed to determine Gaussian distribution and subsequently either One-way-ANOVA or Kruskal-Wallis test was used to determine statistical significance. (D) Displayed is the correlation of CD226, CD161 or CD69 expression with CD16 expression levels on CD56dim NK cells. For CD25, this correlation is also shown for CD56+++ NK cells. Statistical regression analyses are summarized in S2 Table. (TIF) [file pone.0132484.s001.tif]

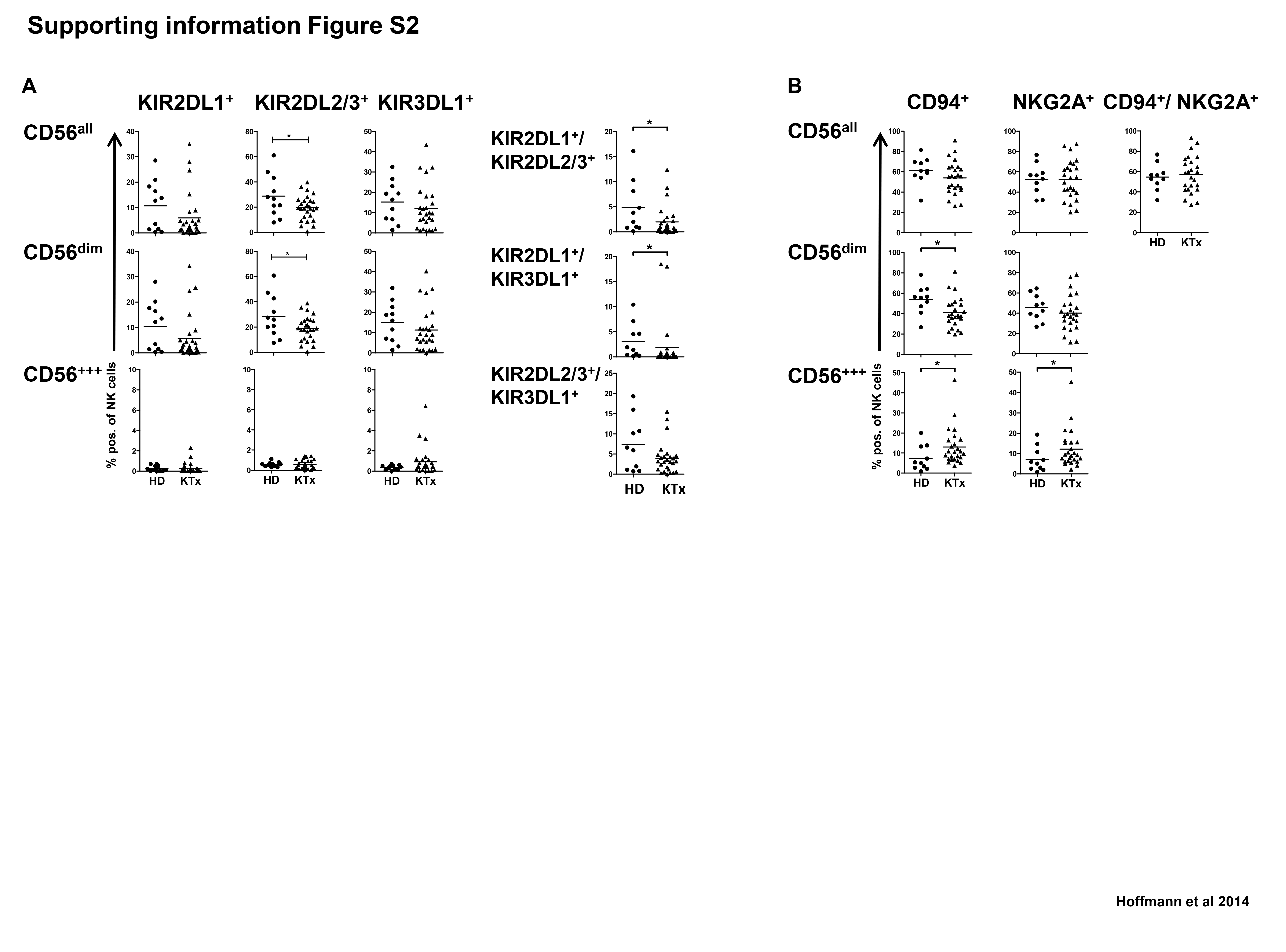

Supplement: S2 Fig — NK cells in PBMC of healthy donors (n = 11, circles) or KTx patients (n = 29, triangles) were analyzed by flow cytometry. (A) Surface expression of KIR2DL1 and 2DS1, KIR2DL2/3 and 2DS2/3 and KIR3DL1 and 3DS1 on all NK cells, CD56dim and CD56bright NK cells as well as the proportion of multiple KIR-positive NK cells was analyzed in HD and compared to KTx patients. Mean values are displayed, D'Agostino & Pearson omnibus normality test was performed to determine Gaussian distribution and subsequently either unpaired, two-sided t test or Mann-Whitney-U test was performed. (B) CD94, NKG2A and CD94/NKG2A surface expression on NK cells of healthy donors and KTR was measured as in C. Data are shown as scatterplots, mean values are displayed. Asterisks indicate the p-values, statistical significance was determined as described in C (* = p≤0.05, ** = p≤0.01 and *** = p≤0.001, only significant values are shown). (TIF) [file pone.0132484.s002.tif]

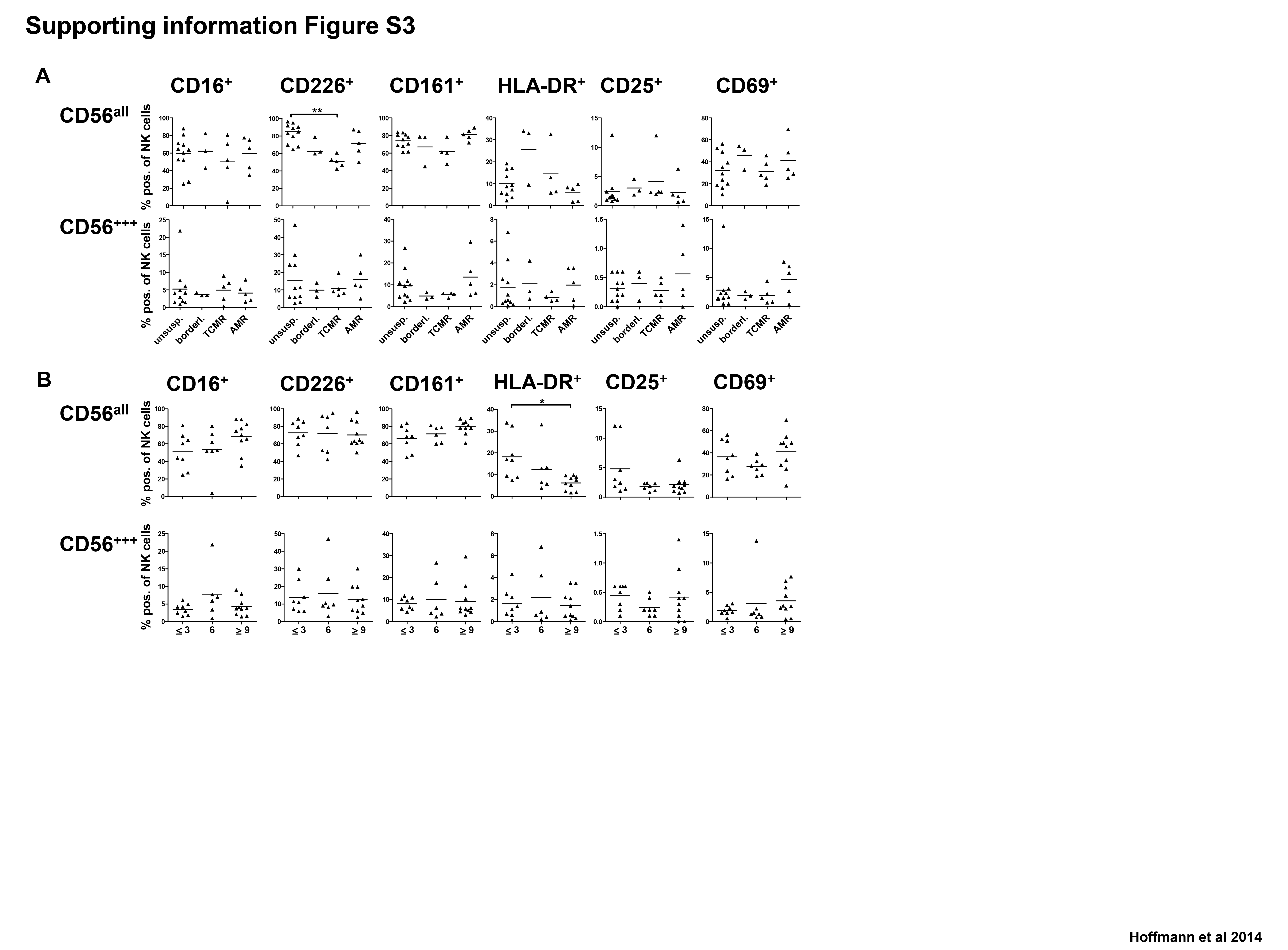

Supplement: S3 Fig — (A) Patients were grouped according to the histopathology of their biopsies: unsuspicious, borderline, TCMR or AMR rejection. Statistical analyses were performed as described for Fig 1B. (B) The impact of time after Tx was determined by grouping patients according to the time interval after Tx: ≤3, 6 or ≥ 9 months. Data are shown as scatter plots and display mean values. Asterisks indicate p-values (* = p≤0.05, ** = p≤0.01 and *** = p≤0.001, only significant values are shown). (TIF) [file pone.0132484.s003.tif]

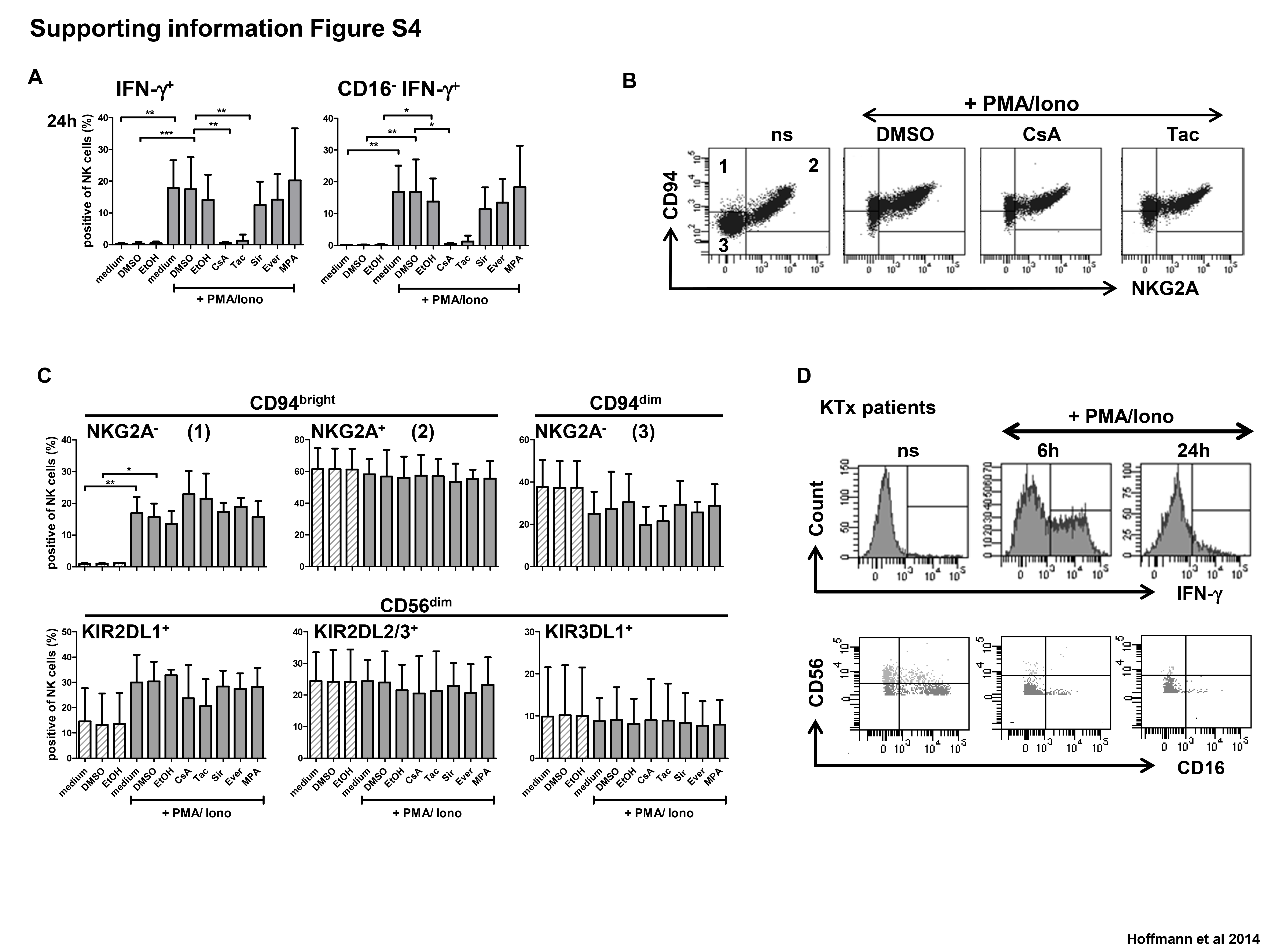

Supplement: S4 Fig — (A) Healthy donor PBMC (n = 6) were pre-incubated with 5μM inhibitor and left either unstimulated (shaded bars) or stimulated with P/I 24h (grey bars). Cells were stained for surface CD3, CD56, CD16 and intracellular IFN-γ. Statistics were performed using Kruskal-Wallis test with Dunn‘s post test (* = p<0.05, ** = p<0.01, *** = p<0.001, only significant values are shown), mean values ± standard deviation are displayed. (B) PBMC from healthy donors (n = 3) were pre-incubated with immunosuppressive drugs or control solvent and stimulated with P/I for 24h or left untreated. NK cells were analyzed for CD94, NKG2A expression by flow cytometry. (C) Statistics were performed for CD94, NKG2A, KIR2DL1/2DS1, KIR2DL2/2DS2, KIR3DL1/2DS1 positive NK cells within the respective subsets using One-way-ANOVA followed by Tukey‘s post test, mean values ± standard deviation are shown (* = p≤0.05, ** = p≤0.01, only significant values are shown). (D) FACS histogram and dot plot analyses are shown for intracellular IFN-γ and surface CD16 expression of gated CD3-CD56+ NK cells either unstimulated or stimulated with P/I for 6h or 24h of one representative KTx patient (ns = non-stimulated). (TIF) [file pone.0132484.s004.tif]

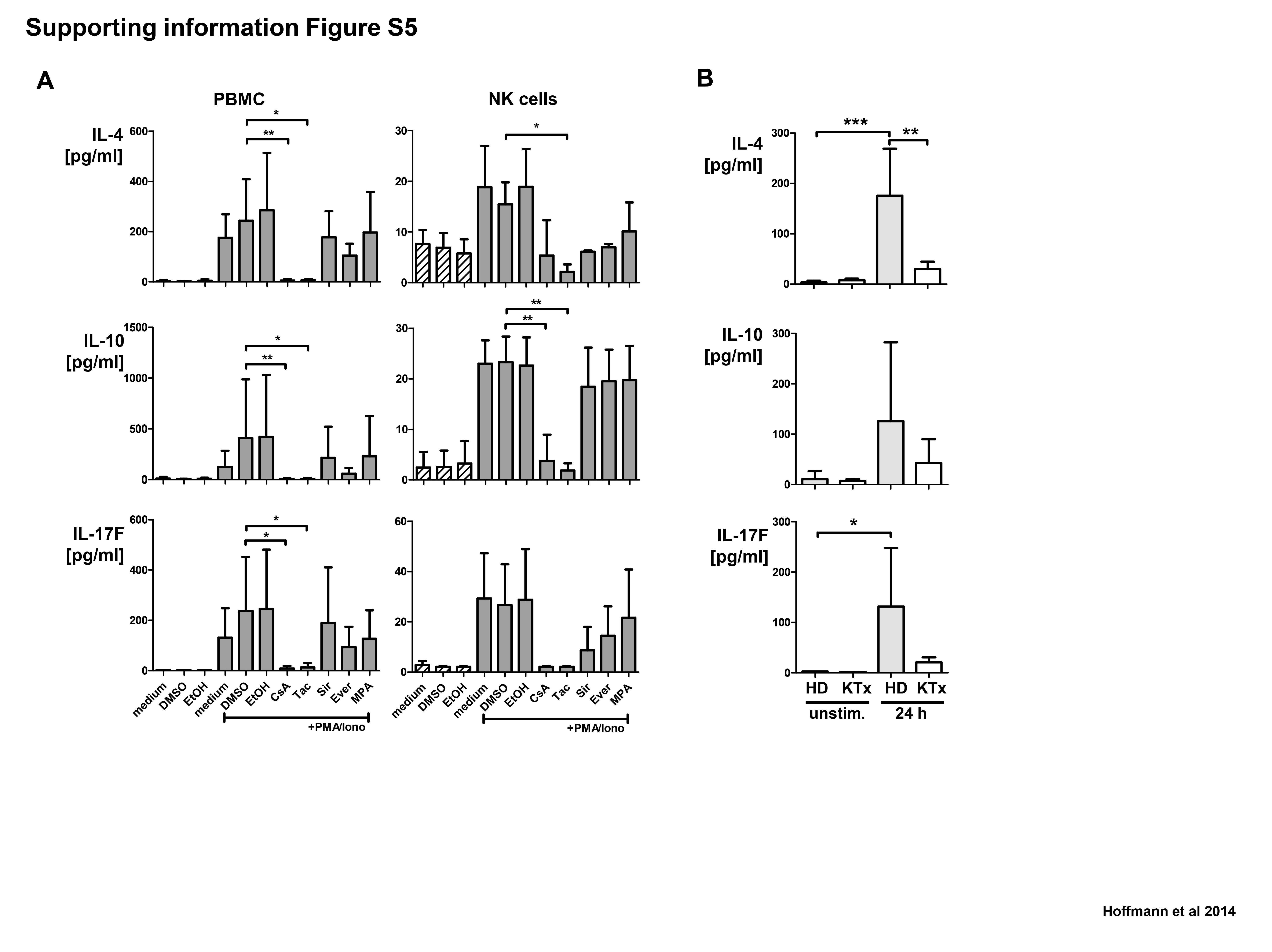

Supplement: S5 Fig — (A) PBMC of healthy donors (n = 6) were pre-incubated for 20 min with 5 μM inhibitor or DMSO solvent, stimulated with P/I for 24h, supernatants were collected and analyzed for IL-4, IL-10 and IL-17F secretion. Mean values ± standard deviation are shown. To determine statistical significance, Kruskal-Wallis test with Dunn’s post test comparing the different inhibitor treatments to DMSO control was performed. NK cells were negatively MACS-isolated from healthy donor PBMC and stimulated as described. To determine statistical significance, One-Way-ANOVA with Dunnett’s Multiple Comparison test was performed (* = p≤0.05, ** = p≤0.01, *** = p≤0.001, only significant values are shown). (B) PBMC of KTx patients (n = 4, white bars) were stimulated for 24h with P/I or left untreated as described, supernatants were collected, tested for IL-4, IL-10 and IL-17F secretion and compared to P/I stimulated PBMCs of healthy donors (n = 6, grey bars). Data are displayed as mean values compared by a two-sided One-way ANOVA with Tukey’s post test (* = p≤0.05, ** = p≤0.01, *** = p≤0.001, only significant values are shown). (TIF) [file pone.0132484.s005.tif]

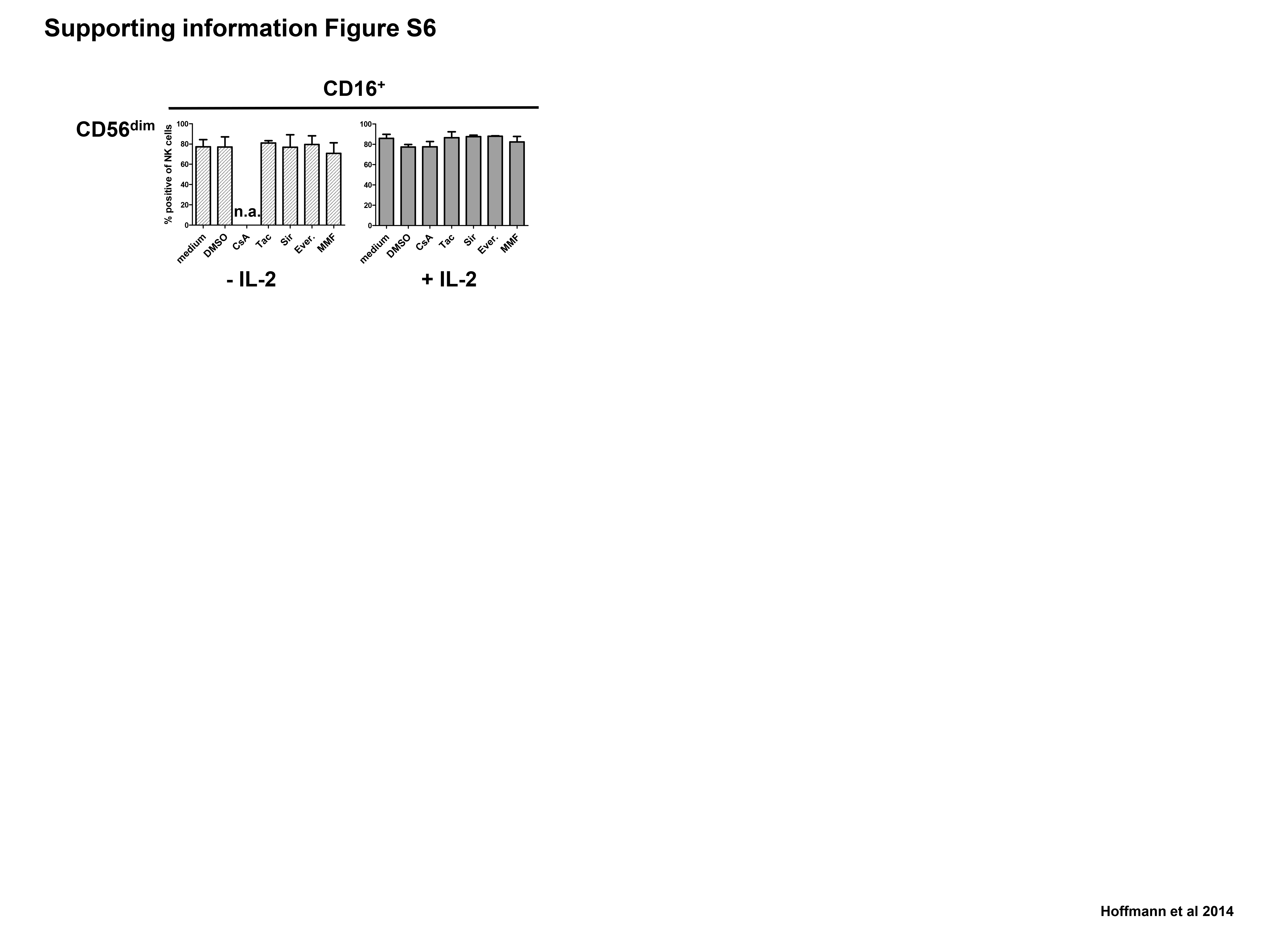

Supplement: S6 Fig — (A) PBMC of healthy donors (n = 4) were incubated for 96h in the presence (grey bars) or absence (shaded bars) of IL-2 and CNI and mTORi treatment, respectively (inhibitor concentration: 10μM). NK cells were stained for CD56 and CD16 and analyzed by flow cytometry. CD16 expression of NK cells treated with CsA alone without addition of IL-2 could not be measured due to toxic effects of this drug on isolated NK cells without IL-2 supplementation. (TIF) [file pone.0132484.s006.tif]
