# Supplementary material for: NK Cells of Kidney Transplant Recipients Display an Activated Phenotype that Is Influenced by Immunosuppression and Pathological Staging
Source: PLoS One. 2015 Jul 6;10(7):e0132484. doi: 10.1371/journal.pone.0132484 (PMC4492590; doi:10.1371/journal.pone.0132484)
Supplement: S1 Table — (DOCX) [file pone.0132484.s007.docx]

**Supporting information Table S1:** mAbs used for cell surface staining

| **antibody** | **clone** | **company** |
| --- | --- | --- |
| CD3-PerCP | (SK7) | Becton-Dickinson, Franklin Lakes, NJ, USA |
| CD94-FITC | (HP3D9) | Becton-Dickinson, Franklin Lakes, NJ, USA |
| HLA-DR-APC | (L243) | Becton-Dickinson, Franklin Lakes, NJ, USA |
| CD226-FITC | (DX11) | Becton-Dickinson, Franklin Lakes, NJ, USA |
| CD45-AmCyan | (2D1) | Becton-Dickinson, Franklin Lakes, NJ, USA |
| p58.1-FITC (KIR2DL1 and S1) | HP-3E4 | Becton-Dickinson, Franklin Lakes, NJ, USA |
| CD16-Pacific Blue | (3G8) | Becton-Dickinson, Franklin Lakes, NJ, USA |
| p58.2-PE (KIR2DL2/3 and S2/3) | GL183 | Beckman-Coulter, Krefeld, Germany |
| CD158.e-ECD (KIR3DL1 and S1) | Z27.3.7 | Beckman-Coulter, Krefeld, Germany |
| CD56-ECD | N901 | Beckman-Coulter, Krefeld, Germany |
| NKG2A-PE | Z199 | Beckman-Coulter, Krefeld, Germany |
| CD16-FITC | 3G8 | Beckman-Coulter, Krefeld, Germany |
| CD161-PE | 191B8 | Beckman-Coulter, Krefeld, Germany |
| CD25-FITC | M-A251 | Beckman-Coulter, Krefeld, Germany |
| CD69-PE | TP1.55.3 | Beckman-Coulter, Krefeld, Germany |
| NKG2D-APC | ON72 | Beckman-Coulter, Krefeld, Germany |
| CD16-APC | 3G8 | Caltag, Buckingham, UK |
