# Supplementary material for: NK Cells of Kidney Transplant Recipients Display an Activated Phenotype that Is Influenced by Immunosuppression and Pathological Staging
Source: PLoS One. 2015 Jul 6;10(7):e0132484. doi: 10.1371/journal.pone.0132484 (PMC4492590; doi:10.1371/journal.pone.0132484)
Supplement: S2 Table — (DOCX) [file pone.0132484.s008.docx]

**Supporting Information Table S2:** statistical evaluation of surface receptor modulation (linear regression)

| **surface markers** | **healthy donors** | | | **KTx patients** | | |
| --- | --- | --- | --- | --- | --- | --- |
|  | r | r² | slope significantly deviant from zero? | r | r² | slope significantly deviant from zero? |
| CD16/CD226 (CD56^dim^ NK cells) | 0.43875 | 0.1925 | no (p=0.2046) | 0.47603 | 0.2266 | yes (p=0.0161) |
| CD16/CD161 (CD56^dim^ NK cells) | 0.23201 | 0.05383 | no (p=0.4924) | 0.55082 | 0.3034 | yes (p=0.0043) |
| CD16/CD69 (CD56^dim^ NK cells) | 0.10756 | 0.01157 | no (p=0.7529) | 0.53122 | 0.2822 | yes (p=0.0052) |
| CD16/CD25 (CD56^dim^ NK cells) | 0.20443 | 0.04179 | no (p=0.5465) | 0.1911 | 0.03652 | no (p=0.3497) |
| CD16/HLA-DR (CD56^dim^ NK cells) | 0.36401 | 0.1325 | no (p=0.2712) | 0.17598 | 0.03097 | no (p=0.4108) |
| CD16/CD25 (CD56^bright^ NK cells) | 0.4443 | 0.1974 | no (p=0.1710) | 0.153 | 0.02341 | no (p=0.4653) |
